# Risk of lymphoma in patients exposed to antitumour necrosis factor therapy: results from the British Society for Rheumatology Biologics Register for Rheumatoid Arthritis

**DOI:** 10.1136/annrheumdis-2016-209389

**Published:** 2017-02-17

**Authors:** Louise K Mercer, James B Galloway, Mark Lunt, Rebecca Davies, Audrey L S Low, William G Dixon, Kath D Watson, BSRBR Control Centre Consortium, Deborah P M Symmons, Kimme L Hyrich

**Affiliations:** 1 Arthritis Research UK Centre for Epidemiology, Centre for Musculoskeletal Research, Manchester Academic Health Science Centre, The University of Manchester, Manchester, UK; 2 Department of Rheumatology, King's College London, London, UK; 3 NIHR Manchester Musculoskeletal Biomedical Research Unit, Central Manchester University Hospitals NHS Foundation Trust and University of Manchester Partnership, Manchester, UK

**Keywords:** Anti-TNF, Epidemiology, Rheumatoid Arthritis

## Abstract

**Objectives:**

Patients with rheumatoid arthritis (RA) are at increased risk of lymphoma compared with the general population. There are concerns that tumour necrosis factor inhibitors (TNFi) may exacerbate this risk. However, since the excess risk of lymphoma in RA is related to the cumulative burden of inflammation, TNFi may conversely reduce the risk of lymphoma by decreasing the burden of inflammation. The aim of this study was to compare the risk of lymphoma in subjects with RA treated with TNFi with those treated with non-biological therapy.

**Methods:**

Subjects diagnosed by a rheumatologist with RA enrolled in the British Society for Rheumatology Rheumatoid Arthritis Register (BSRBR-RA), a prospective cohort study, were followed until first lymphoma, death or until 30 November 2013. Rates of lymphoma in the TNFi and non-biological-treated cohorts were compared using Cox regression.

**Results:**

11 931 TNFi-treated patients were compared with 3367 biological-naive patients. 84 lymphomas (88 (95% CI 70 to 109) per 100 000 person-years) were reported in the TNFi cohort and 30 lymphomas (154 (95% CI 104 to 220)) in the biological-naive cohort. After adjusting for differences in baseline characteristics, there was no difference in the risk of lymphoma for the TNFi versus the biological-naive group: HR 1.00 (95% CI 0.56 to 1.80). No risk differences were observed for individual TNFi.

**Conclusions:**

In medium-term follow-up, there is no evidence that tumour necrosis factor inhibition influences the risk of lymphoma over the background risk in subjects with RA.

## Introduction

In the late 1990s, the treatment of rheumatoid arthritis (RA) and other related autoimmune inflammatory conditions underwent a fundamental shift, away from general immunosuppressive agents towards an approach that targeted specific components of the inflammatory pathway. The first treatments in this therapeutic class, known collectively as biological agents, were inhibitors of tumour necrosis factor-alpha (TNF-alpha).^1–3^ Tumour necrosis factor (TNF) plays a pivotal role in inflammation in RA^4^ and tumour necrosis factor inhibitors (TNFi) are highly effective in treating RA.^5^ From early in their development, there were concerns regarding the long-term safety of the TNFi with respect to malignancy, and in particular lymphoma.^6^ ^7^ The possible effects of TNF inhibition on lymphomagenesis are difficult to predict. TNF has pleotropic effects in the promotion and progression of malignancy, with both tumour-promoting and tumour-inhibiting actions.^8^ One of the main indications for anti-TNF therapies is RA and RA itself has a long-recognised established increased risk of lymphoma compared with the general population,^9^ ^10^ especially the diffuse large B cell lymphoma (DLBCL).^11^ ^12^ Importantly, a large Swedish nested case–control study reported that patients in the highest decile of cumulative RA disease activity had more than a 60-fold increased risk of lymphoma compared with those in the lowest decile (OR 61.6 (95% CI 21.0 to 181.1)).^13^ A previous publication from the British Society for Rheumatology Rheumatoid Arthritis Register (BSRBR-RA) demonstrated that there remains an increased risk of lymphoma in biological-naïve patients treated with non-biological therapy compared with the general population in the modern era of early and aggressive treatment.^14^ There is some evidence that this increased risk in RA may be exacerbated further by immunosuppressive therapy.^15^ Therefore, given the strong association between chronic inflammation and lymphoma development in RA, it is plausible that TNFi could reduce the risk of lymphoma by reducing ongoing inflammation. Nonetheless, the TNFi carry a black box warning with respect to lymphoma and the US Food and Drug Administration have highlighted concerns about the risk of hepatosplenic T cell lymphoma, a rare and aggressive cancer, in children and adolescents.^7^

Several European biological registers have been established over the last 10–15 years to examine the long-term safety of TNFi.^16^ One of the earliest and largest of these, the BSRBR-RA, was established in 2001 with a primary aim to determine the relationship between exposure to TNFi and lymphoma risk.^17^ Here, we report the risk of lymphoma development in patients with RA exposed to TNFi therapy and compare that with the risk in patients with RA treated with non-biological (synthetic) disease-modifying drug (csDMARD) therapy.

## Methods

### Patients

Subjects were participants in the BSRBR-RA, an ongoing national prospective observational cohort study established in 2001 to monitor the long-term safety of biological therapy in RA. UK national guidelines from the National Institute for Health and Care Excellence (NICE) recommend that prescription of TNFi is restricted to patients with highly active disease.^18^ ^19^ This is defined as a score >5.1 using the 28-joint Disease Activity Score (DAS28)^20^—a composite score of swollen and tender joint counts, erythrocyte sedimentation rate and a patient's global assessment of disease—despite treatment with at least two csDMARDs, one of which should be methotrexate.^18^ ^19^ During the time period of recruitment of patients included in this analysis, three TNFi agents were available in the UK: etanercept (ETA), infliximab (INF) and adalimumab (ADA). A comparison cohort of biological-naïve patients with RA was recruited in parallel and followed in an identical manner to the TNFi cohort.^17^ These patients had active disease at recruitment (target DAS28≥4.2) despite current treatment with csDMARD. The subjects' written consent was obtained.

#### BSRBR-RA data collection methods

Baseline data for all patients, collected via rheumatologist/nurse-completed questionnaire, included demographics, disease duration, disease activity, current and past csDMARDs, baseline corticosteroid use, comorbidities and smoking history. Patients completed a Stanford Health Assessment Questionnaire (HAQ) adapted for British use^21^ to indicate the level of physical disability and were asked to select their ethnic group from a list: white; black-African; black-Caribbean; black-British; black-other; Indian; Pakistani; Bangladeshi; Chinese or other (please specify). All patients were flagged at baseline with the National Health Service Information Centre or the Northern Ireland Cancer Registry, who link with the British cancer agencies. The cancer registries notified the BSRBR-RA of past cancers and prospectively of cancers that occurred after the patient entered the register. Capture of cancer cases is very high using these sources, for example, 97% for England cancer statistics published for 2009.^22^

All patients were continually followed until death or self-withdrawal, regardless of changes to antirheumatic therapies. Changes to RA therapy were reported on rheumatologist/nurse questionnaires completed 6-monthly for 3 years then annually thereafter. Data on adverse events were captured in three ways: from rheumatologist/nurse questionnaires; from 6-monthly patient diaries completed for 3 years on which details of any new hospital admissions, physician consultations and treatments were recorded and by flagging with the national death register and cancer registries which reported malignancies using the 10th edition of the International Classification of Diseases (ICD-10). Additional information (including histology) was sought from rheumatologist for all incident lymphomas.

#### Outcome

The primary outcome measure for this analysis was first verified lymphoma per subject. Lymphomas were verified if they fulfilled either of the following criteria: (i) confirmation on histology report or (ii) reported by a national cancer agency. There were no lymphomas identified from death certificates alone. Histology reports and ICD-10 codes reported by the cancer registries were used to classify lymphomas into subtypes.

#### Subject selection for the current analysis

**Figure 1:**
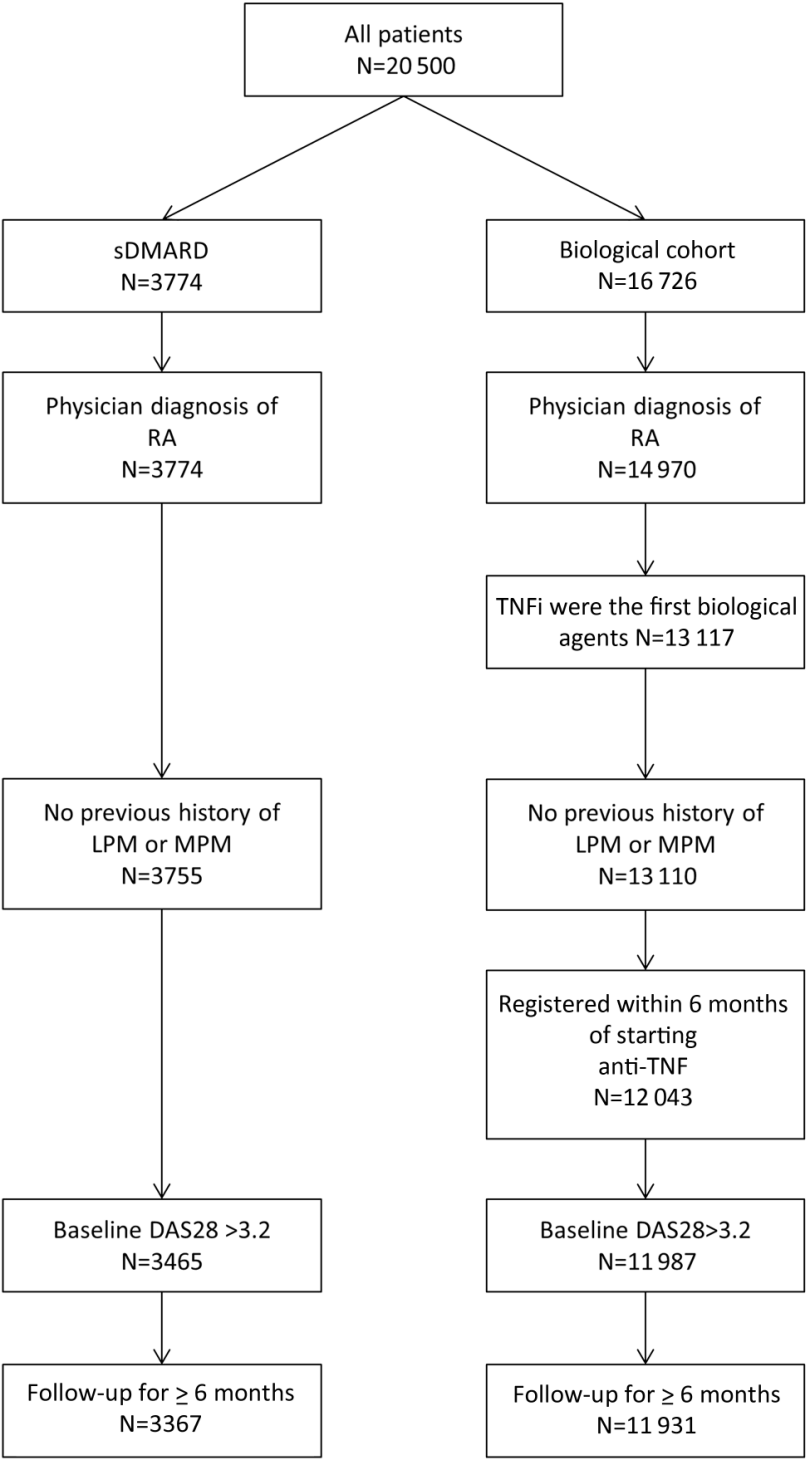
Selection of participants for the analysis. DAS28, 28-joint Disease Activity Score; LPM, lymphoproliferative malignancy; MPM, myeloproliferative malignancy; RA, rheumatoid arthritis; TNF, tumour necrosis factor; TNFi, tumour necrosis factor inhibitors.

Stata (StataCorp., College Station, Texas, USA) V.12.1 was used for the analyses. Patients were selected from the register if they had a physician diagnosis of RA and at least one returned rheumatologist follow-up questionnaire prior to 30 November 2013. Patients with a prior diagnosis of lymphoproliferative or myeloproliferative malignancy (LPM/MPM) were excluded from the analysis (figure 1). The TNFi cohort was restricted to patients who received ETA, INF or ADA as their first biological therapy and who registered with the BSRBR-RA within 6 months of starting treatment. Patients with prior biological exposure were excluded. The first 6 months of follow-up time was excluded from both cohorts, to minimise the risk of including prevalent lymphomas in the analysis, and so patients who did not complete 6 months follow-up were excluded. Patient-years (pyrs) of follow-up time were calculated from 6 months after the date of starting a TNFi or 6 months after the date of registration with the BSRBR-RA for the csDMARD cohort. Follow-up was censored at the date of first lymphoma, death or on 30 November 2013, whichever came first. Patients in the csDMARD cohort who subsequently started a biological drug contributed follow-up time up until the date of first dose biological therapy. Follow-up time after stopping TNFi was included in the TNFi cohort, irrespective of whether or not the patient started a second or subsequent biological drug, since it was hypothesised that the effects of TNFi on lymphoma risk may be long-lasting.

### Statistical analysis

Baseline characteristics of the cohorts were compared using the χ^2^ test for categorical variables and the Wilcoxon rank-sum test for continuous items and p values were presented. Rates of lymphoma are presented as total events per 100 000 pyrs with 95% CIs constructed assuming a Poisson distribution of cases. Risk comparisons were made between the TNFi and csDMARD cohorts using Cox regression. Potential confounders were identified a priori and comprised age, sex, smoking status and ethnicity, since the incidence of lymphoma varies by ethnic group^23^ (dichotomised as white or other ethnicity due to the high proportion of white participants); prior cancer (excluding LPM or MPM); comorbidity and markers of RA severity. The number of comorbidities was calculated as a composite variable constructed from the following conditions: hypertension; ischaemic heart disease; stroke; asthma, bronchitis or emphysema; renal disease; diabetes mellitus; liver disease and depression. Several baseline dimensions of RA disease severity were included: DAS28 score; HAQ score; RA duration; number of previous csDMARDs (categorised as ≤3 or ≥4) and current exposure to corticosteroids. Date of registration with the BSRBR-RA (dichotomised as before/after June 2004) was also included to account for unmeasured confounding relating to temporal changes in the way that rheumatologists managed patients with RA.

Adjustment for these covariates was performed by calculating a propensity score (PS) that reflected the likelihood that an individual patient was prescribed a TNFi, given the above characteristics. The PS has a single value for each subject that can be substituted into the regression model in place of all of the potentially confounding covariates. The PS model was derived and tested using ‘*Prop_sel*’ commands in Stata. The balance of the model was tested by examining the expected bias, which is the likely bias in the treatment estimate due to each confounder. A maximum bias of 2% in either direction was considered acceptable (see online supplementary figure S1). The PS was stratified into deciles of propensity score (PD) for use in adjusted analyses. Subjects with a baseline DAS28 score ≤3.2 (csDMARD: 290; TNFi: 56) were subsequently excluded from the analysis due to an area of non-overlap in the PS between cohorts. Missing baseline data were replaced using multiple imputation (see online supplementary table S1). The assumptions of the Cox models were tested using Schoenfeld residuals and met.

10.1136/annrheumdis-2016-209389.supp1 Supplementary data

#### Drug exposure models

The primary analysis compared the rate of lymphoma in patients ever treated with TNFi with the csDMARD cohort (ie, subjects in the TNFi cohort were considered exposed from first dose until the end of follow-up—ever exposed analysis). Additional analyses were performed to test the robustness of the findings. First, patients actively on TNFi (including a lag period of 90 days from their first missed dose) were compared with the csDMARD cohort (on-drug analysis). For on-drug analyses, time after last received consultant follow-up questionnaire was excluded for all participants since information about current drug exposure status came from these questionnaires. Second, the outcome was restricted to lymphomas reported by the national cancer agencies, in case there was any bias in reporting of lymphomas by patients and rheumatologists to the BSRBR-RA. Lymphoma subgroup-specific analyses were performed for subtypes with ≥5 lymphomas in each cohort as a secondary outcome.

The risk of first lymphoma for each TNFi agent was compared separately with csDMARD. Propensity models for each TNFi versus csDAMRD were created using the same method and confounders as above. Three drug exposure models were used. First, lymphomas were attributed to the most recently received TNFi. Second, lymphomas were attributed to the first TNFi agent received and follow-up was censored when a second biological drug was started and third, an on-drug model.

## Results

In total, 3367 csDMARD and 11 931 TNFi patients were included (figure 1). The TNFi cohort comprised 4144 (35%) patients starting ETA; INF 3499 (29%) and ADA 4288 (36%). The TNFi cohort was younger and comprised more women (table 1). As might be expected, the TNFi cohort had more severe disease, of longer duration and greater exposure to corticosteroids and prior csDMARD. The median follow-up was 6.5 years (IQR 3.8, 8.0) for csDMARD and 8.6 (6.7, 9.7) for TNFi.

**Table 1:** Baseline characteristics

	csDMARD n=3367	All TNFi n=11 931	p Value
Mean age: years (SD)	60 (12)	56 (12)	<0.001
Females: %	2477 (74)	9115 (76)	<0.001
Smoking history n (%)			
Current smoker	795 (24)	2595 (22)	0.001
Former smoker	1335 (40)	4530 (38)	
Never smoked	1222 (36)	4727 (40)	
Not recorded	15 (0)	79 (1)	
Ethnicity n (%)			
White	2561 (76)	9848 (83)	<0.001
Other	64 (2)	410 (3)	
Not recorded	742 (22)	1673 (14)	
Mean DAS28 (SD)	5.3 (1.1)	6.6 (0.9)	<0.001
Mean HAQ (SD)	1.5 (0.7)	2.0 (0.6)	<0.001
Median disease duration: years (IQR)	6 (1, 15)	11 (6 19)	<0.001
Baseline steroid use: n (%)	759 (23)	5259 (44)	<0.001
Number of prior csDMARD: median (IQR)	2 (1, 3)	4 (3, 5)	<0.001
Comorbidity*: n (%)			
None	1397 (41)	5530 (46)	<0.001
1 comorbidity	1163 (35)	4098 (34)	
2 comorbidities	560 (17)	1691 (14)	
≥3 comorbidities	247 (7)	612 (5)	
Prior solid cancer: n (%)	122 (4)	170 (1)	<0.001

*Hypertension, ischaemic heart disease (myocardial infarction or angina), stroke, lung disease (asthma, bronchitis or emphysema), diabetes mellitus, depression, renal disease and liver disease.

csDMARD, non-biological (synthetic) disease-modifying drug; DAS28, 28-joint Disease Activity Score; HAQ, Health Assessment Questionnaire; TNFi, tumour necrosis factor inhibitors.

Seven lymphomas were diagnosed in the first 6 months and not included (csDMARD 3, TNFi 4). One hundred and fourteen lymphomas were diagnosed during subsequent follow-up (30 in 19 473 pyrs in the csDMARD and 84 in 95 126 pyrs in the TNFi cohort (table 2)). The proportion of lymphomas reported by the national cancer registries was 90% in the csDMARD and TNFi cohorts (table 2). The unadjusted HR for lymphoma for TNFi compared with csDMARD was 0.61 (95% CI 0.40 to 0.92) (table 3). Age and male gender were associated with the risk of lymphoma in univariate analysis (see online supplementary table S2). After fully adjusting, using PD, there was no difference in the risk of lymphoma for TNFi compared with csDMARD; HR 1.00 (95% CI 0.56 to 1.80) (table 3). The PD-adjusted HR for the ‘on TNFi’ analysis was 1.17 (95% CI 0.60 to 2.26). Excluding time after starting second biological drug or limiting the analysis to cancer registry-only reported lymphomas did not materially alter the findings (table 3). There were five Hodgkin's lymphomas (HL; 17%) and 25 non-Hodgkin's lymphomas (NHL; 83%) in the csDMARD cohort; and 12 HL (14%) and 72 NHL (86%) in the TNFi cohort (table 2). The most frequently reported subtype of NHL was DLBCL (table 2). No significant differences in the proportion of HL, NHL or DLBCL were seen between cohorts (table 3). Five T cell lymphomas were reported, none of which were the hepatosplenic subtype.

**Table 2:** Characteristics of lymphomas

	csDMARD N=3367	TNFi N=11 931	Most recent TNFi		
			Adalimumab N=4288	Etanercept N=4144	Infliximab N=3499
Total follow-up time (pyrs)	19 473	95 126	33 354	40 619	21 150
Median follow-up per subject (IQR)	6.5 (3.8, 8.0)	8.6 (6.7, 9.7)	6.2 (3.5, 8.0)	5.9 (1.4, 9.1)	8.1 (3.8, 9.2)
Lymphomas	30	84	34	29	21
Sources of reporting of lymphomas					
Cancer registry (%)	27 (90)	76 (90)	30 (88)	25 (86)	21 (100)
Subtypes of lymphoma: N (%)					
HL	5 (17)	12 (14)	4 (12)	5 (17)	3 (14)
NHL					
DLBCL	10 (33)	31 (37)	19 (56)	5 (17)	7 (33)
FL	1 (3)	18 (21)	5 (15)	6 (21)	7 (33)
CLL/small lymphocytic	3 (10)	8 (9)	2 (6)	4 (14)	2 (10)
MALToma	0	4 (5)	1 (3)	3 (10)	0
Mantle cell	3 (10)	0	0	0	0
Burkitt	1 (3)	0	0	0	0
B cell NHL NOS	5 (17)	8 (9)	2 (6)	4 (14)	2 (10)
T cell	2 (7)	3 (4)	1 (3)	2 (7)	0

CLL, chronic lymphocytic leukaemia; DLBCL, diffuse large B cell lymphoma; FL, follicular lymphoma; HL, Hodgkin's lymphoma; MALToma, mucosal-associated lymphoid tissue lymphoma; NHL, non-Hodgkin's lymphoma; NOS, not otherwise specified; pyrs, patient-years; TNFi, tumour necrosis factor inhibitors.

**Table 3:** Association between exposure to TNFi and lymphoma

	csDMARD N=3367	TNFi N=11 931
Total follow-up time (pyrs)	19 473	95 126
Lymphomas	30	84
Incidence rate per 100 000 pyrs (95% CI)	154 (104 to 220)	88 (70 to 109)
Unadjusted HR (95% CI)	Referent	0.61 (0.40 to 0.92)
Age-adjusted and sex-adjusted HR (95% CI)	Referent	0.75 (0.49 to 1.15)
PD-adjusted HR (95% CI)	Referent	1.00 (0.56 to 1.80)
On TNFi (plus 90 days)*		
Follow-up time (pyrs)	15 167	57 949
Lymphomas	25	63
PD-adjusted HR (95% CI)	Referent	1.17 (0.60 to 2.26)
Excluded time after switched to second biological drug*		
Follow-up time (pyrs)	15 167	55 167
Lymphomas	25	52
PD-adjusted HR (95% CI)	Referent	1.12 (0.58 to 2.18)
Cancer registry-only reported lymphomas		
Follow-up time (pyrs)	19 473	95 126
Lymphomas	27	76
PD-adjusted HR (95% CI)	Referent	1.02 (0.55 to 1.90)
Hodgkin's lymphomas (HL)		
Incidence rate of HL per 100 000 pyrs (95% CI)	26 (8 to 60)	13 (7 to 22)
PD-adjusted HR for HL (95% CI)	Referent	0.54 (0.12 to 2.50)
Non-Hodgkin's lymphomas (NHL)		
Incidence rate of NHL per 100 000 pyrs (95% CI)	128 (83 to 190)	75 (58 to 94
PD-adjusted HR for NHL (95% CI)	Referent	1.10 (0.58 to 2.08)
DLBCL		
Incidence rate of DLBCL per 100 000 pyrs (95% CI)	67 (36 to 114)	56 (42 to 73)
PD-adjusted HR for DLBCL (95% CI)	Referent	1.54 (0.60 to 3.95)

*Time after last received consultant follow-up form excluded from this analysis.

DLBCL, diffuse large B cell lymphoma; pyrs, patient-years; TNFi, tumour necrosis factor inhibitors.

Thirty-four lymphomas were reported in patients most recently exposed to ADA, 29 to ETA and 21 to INF. No difference in the relative risk of lymphoma was seen for any TNFi compared with csDAMRD using each of the exposure models (table 4).

**Table 4:** Association between exposure to adalimumab (ADA), etanercept (ETA) or infliximab (INF) and lymphoma

	ADAN=4288	ETA N=4144	INF N=3499
First TNFi received (censored when second biological drug started)*			
Total follow-up time (pyrs)	22 361	26 838	17 688
Number of lymphomas	20	20	18
Incidence rate per 100 000 pyrs (95% CI)	89 (55 to 138)	75 (45 to 115)	102 (60 to 161)
PD-adjusted HR (95% CI) (csDMARD referent)	1.00 (0.49 to 2.03)	1.02 (0.45 to 2.33)	0.91 (0.39 to 2.13)
Most recently received TNFi			
Follow-up time (pyrs)	33 354	40 618	21 149
Number of lymphomas	34	29	21
Incidence rate per 100 000 pyrs (95% CI)	102 (71 to 143)	71 (48 to 103)	99 (62 to 152)
PD-adjusted HR (95% CI) (csDMARD referent)	0.99 (0.52 to 1.88)	0.78 (0.37 to 1.66)	0.82 (0.37 to 1.82)
On drug (plus 90 days)*†			
Follow-up time (pyrs)	18 818	24 984	12 328
Number of lymphomas	23	10	10
Incidence rate per 100 000 pyrs (95% CI)	122 (77 to 183)	40 (19 to 74)	81 (39 to 149)
PD-adjusted HR (95% CI) (csDMARD referent)	0.77 (0.37 to 1.61)	0.41 (0.14 to 1.19)	0.75 (0.27 to 2.09)

*Time after last received consultant follow-up form excluded from these analyses.

†Includes both first and subsequent exposures to the drug.

pyrs, patient-years; TNFi, tumour necrosis factor inhibitors.

## Discussion

This national prospective study, with over 120 000 pyrs of exposure to anti-TNF or csDMARD, did not identify any difference in lymphoma risk for up to 8 years after the addition of TNFi to the standard treatment regimen of patients with RA.

This study represents one of the largest and most detailed analyses of TNFi and lymphoma risk published until now and mirrors the results of previous cohort studies^24–27^ and meta-analyses.^28–30^ The question of whether or not anti-TNF influences the risk of lymphoma is of particular concern to rheumatologists due to the known association between severity of RA and lymphoma^13^ and a signal for increased cancer risk following TNF inhibition in an early meta-analysis.^31^ A clinically meaningful increased risk of lymphoma associated with TNFi was excluded from this analysis: the analysis had 98% power to detect a twofold relative increased risk for TNFi compared with the rate in the csDMARD cohort.

A further strength of the current study was that only incident users of TNFi as their first biological drug for RA were included. Furthermore, data on lymphomas in our study were collected by flagging all participants with the UK cancer agencies that have near-complete capture of cases, thus minimising potential for bias in reporting between cohorts. Further information was requested, including histology reports, for all reported cancers, ensuring that a standard data set was received for each cancer.

The selection of an ‘ever exposed’ to TNFi drug model reflects the hypothesis that any effect of TNFi on lymphoma risk would be long-lasting and may operate in the latent period of a cancer. Alternative exposure models were constructed, which did not materially change the findings.

Other strengths of this study include careful consideration of potential biases. For example, the first 6 months of follow-up were excluded to reduce the probability of prevalent lymphomas being included. In addition, prevalent users of TNFi at baseline were excluded. The analysis adjusted for a wider range of confounders than previous studies, as confounding by indication is likely to be present given the way these agents are currently prescribed for RA. Differences in the baseline characteristics of the cohorts reflect this to some degree. The csDMARD cohort was older and comprised more men than the TNFi cohort; both risk factors for cancer, which may explain in part the lower crude risk of lymphoma in the TNFi cohort. Despite lymphoma being a relatively uncommon outcome, adjustment for multiple confounders was possible by using PS methods. Stratifying the PS into deciles reduced the expected bias in the analysis to less than 5%.

The results of this study align with those of previous analyses of lymphoma in users of anti-TNF. Wolfe and Michaud^25^ used data from the National Data Bank and reported an OR for exposure to TNFi of 1.0 (95% CI 0.6 to 1.8). However, this study included prevalent users of TNFi, leading to possible left censorship, unlike our study. The Swedish Biologics Register (Anti-Rheumatic Therapies in Sweden; ARTIS) reported a relative risk of 1.35 (95% CI 0.82 to 2.11) in their most recent publication.^24^ However, despite having more than 350 000 pyrs of follow-up in their csDMARD comparator cohort, they lacked precision in their estimate of drug effect since they observed only 26 lymphomas in 30 000 pyrs in the TNFi cohort.

The French Research Axed on Tolerance of bIOtherapies (RATIO) registry reported that exposure to ADA or INF versus ETA was a risk factor for lymphoma over a 3-year period (OR 4.7 (1.3 to 17.7) and 4.1 (1.4 to 12.5), respectively).^32^ Conversely, this current study did not observe a difference in the risk of lymphoma for any individual TNFi versus csDMARD. Direct comparisons were not made in this study since more than 50% of the cohort were exposed to multiple TNFi during follow-up. Such patients who switch drugs, commonly for treatment inefficacy, may have a different underlying risk of lymphoma.

The most frequently reported subtype in this study was DLBCL, with follicular lymphoma being the next most frequent subtype in the TNFi cohort, in line with previous studies of biological-naive cohorts.^11^ ^13^ The distribution of lymphoma subtypes varies by sex and age at diagnosis and so the difference in age between the TNFi and csDMARD cohorts may have led to a difference in the expected distribution of subtypes between groups. The proportion of follicular lymphomas was lower in the csDMARD cohort than expected but the numbers of individual subtypes were low, highlighting the fact that this study was not powered to study at the relative risk of individual subtypes. Reporting of lymphoma subtypes was based on external histology reports rather than rereview of lymphoma specimens and misclassification between lymphoma subtypes may have occurred.

A weakness of our study was that it was not possible to adjust for, or explore the influence of, cumulative RA disease activity. However, HAQ, a marker of cumulative damage, baseline DAS28 and RA duration were included in the PS model. Missing data can adversely affect all studies, particularly observational studies. Overall, the proportion of missing baseline data was low in the BSRBR-RA (see online supplementary material). Response rates to follow-up questionnaires were excellent; less than 1% of patients in each cohort had no returned consultant follow-up. To minimise bias introduced by missing baseline data, multiple imputation was used.

In conclusion, this study has ruled out an important risk of lymphoma in patients with RA exposed to TNFi over the background risk associated with RA for up to 5 years after treatment initiation. This is consistent with other published data and the biological expectation that disease activity is the primary driver for lymphoma in RA. Further follow-up of significantly larger populations is now needed to determine whether longer-term exposure or cumulative drug exposure influences risk, given the overall low absolute risk of lymphoma in TNFi-treated patients.
