# Supplementary material for: Risk of lymphoma in patients exposed to antitumour necrosis factor therapy: results from the British Society for Rheumatology Biologics Register for Rheumatoid Arthritis
Source: Ann Rheum Dis. 2017 Feb 17;76(3):497–503. doi: 10.1136/annrheumdis-2016-209389 (PMC5446004; doi:10.1136/annrheumdis-2016-209389)
Supplement: Supplementary data [file annrheumdis-2016-209389supp001.pdf]

ONLINE-ONLY SUPPLEMENTARY MATERIAL

Statistical metholodology supplementary information

Multiple imputations were performed in Stata version 12.1 using the ICE command. Missing data were present in the following variables: smoking, ethnicity, RA disease duration, baseline HAQ, and baseline DAS28 score (Table S1). The imputation model was constructed separately for the csDMARD and TNFi cohorts. Age, sex, disease duration, baseline HAQ, baseline DAS28 score and separate components (28 swollen joint count, 28 tender joint count, patient global visual analogue score and ESR), baseline steroid exposure, baseline non-steroidal anti-inflammatory exposure, prior csDMARD use, smoking status, entry year, co-morbidity, prior cancer, ethnicity, height and weight were all included as predictors within the imputation model. The outcome (lymphoma) was also included in the model. Twenty imputation cycles were performed and the resulting data were analysed using Rubin's rules with the MIM command.

Table S1. Missing baseline data

Variable; N missing (%)	csDMARD<br>N=3367	TNFi<br>N=11931
Disease duration	22 (1)	90 (1)
HAQ score	695 (21)	589 (5)
DAS 28 score	53 (1)	110 (1)
Smoking	15 (0)	77 (1)
Ethnicity	745 (22)	1653 (14)

Table S2. Age and sex-adjusted hazard ratios for lymphoma (ever exposed to TNFi model)

	**Hazard ratio (95% CI) for covariate**	**Hazard ratio for TNFi (95% CI)**
Unadjusted HR for TNFi		0.61 (0.40, 0.92)
Age (sex adjusted)	1.04 (1.02, 1.06)	0.75 (0.49, 1.15)
Sex (age adjusted; male referent)	0.52 (0.36, 0.77)	0.75 (0.49, 1.15)
Ethnicity (white referent)	0.76 (0.28, 2.08)	0.75 (0.49, 1.15)
Smoking (Current smoker referent):<br>Ex-smoker<br>Never smoked	 1.15 (0.70, 1.89)<br>0.78 (0.45, 1.35)	 0.75 (0.49, 1.15)
Prior cancer*	1.61 (0.59, 4.41)	0.76 (0.50, 1.17)
Comorbidity (Nil referent):<br>1 comorbidity<br>2 comorbidities<br>≥3 comorbidities	 0.77 (0.51, 1.18)<br>0.88 (0.52, 1.49)<br>0.35 0.11. 1.13)	 0.75 (0.49, 1.14)
Entered study before June 2004	1.11 (0.76, 1.64)	0.73 (0.47, 1.14)
Disease duration (per year)	1.00 (0.98, 1.02)	0.76 (0.49, 1.18)
Disease activity (DAS28)	0.92 0.77, 1.11)	0.84 0.51, 1.39)
Disability (HAQ)	0.93 (0.68, 1.28)	0.78 (0.49, 1.24)
Baseline corticosteroids	0.84 (0.57, 1.24)	0.78 (0.51, 1.21)
No. prior csDMARD	1.06 (0.72, 1.56)	0.74 (0.47, 1.16)

* Patients with prior lymphoproliferative or myeloproliferative malignancy were excluded

Figure S1. Expected bias before and after adjustment using the PS

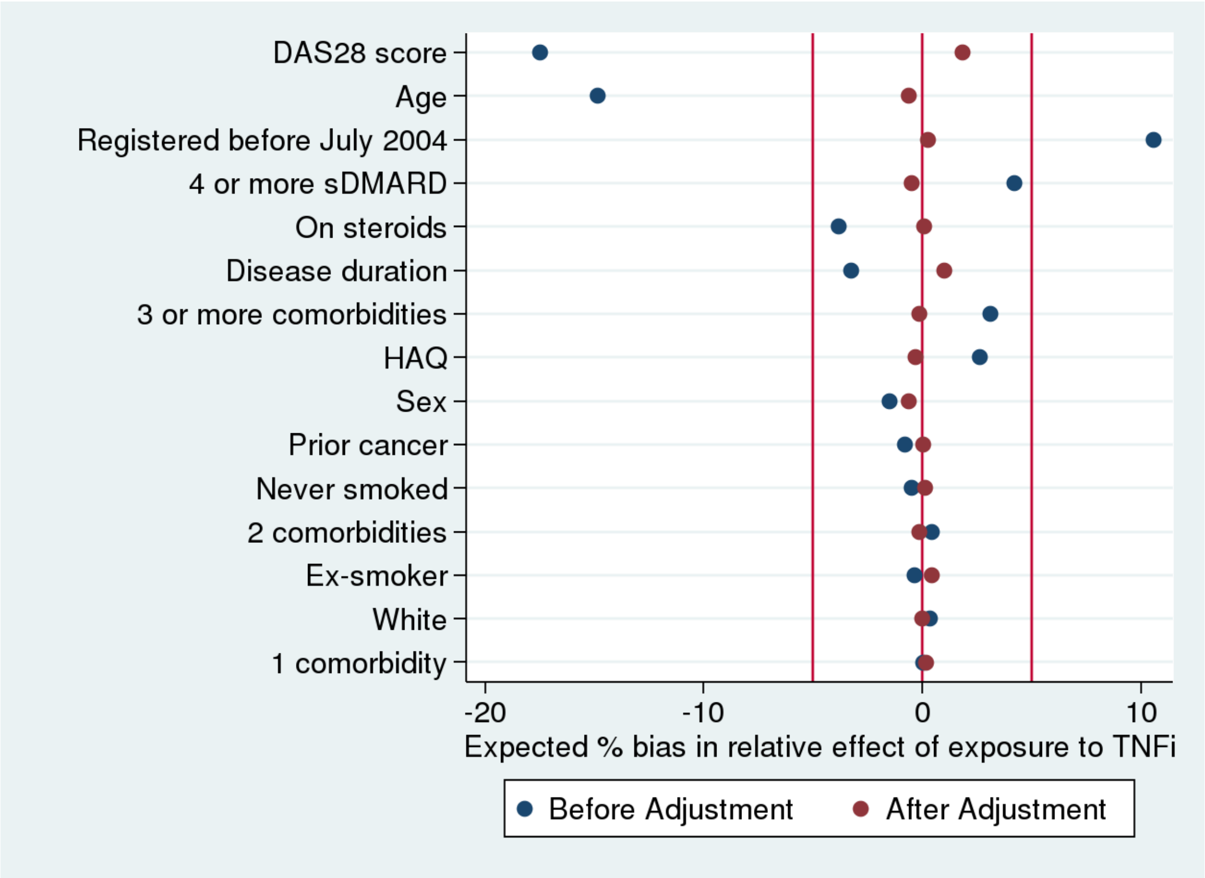

Components of the PS model were: age, sex, ethnicity; co-morbidity; DAS28 score; HAQ score; RA duration; smoking status; number of previous csDMARDs; exposure to corticosteroids; and date of registration with the BSRBR-RA (dichotomised as before/after June 2004). Additional terms comprised the square of DAS28 score, the cube of DAS28 score and an interaction term between age and DAS28 score.
